# Environmental STEM Study of the Oxidation Mechanism for Iron and Iron Carbide Nanoparticles

**DOI:** 10.3390/ma15041557

**Published:** 2022-02-18

**Authors:** Alec P. LaGrow, Simone Famiani, Andreas Sergides, Leonardo Lari, David C. Lloyd, Mari Takahashi, Shinya Maenosono, Edward D. Boyes, Pratibha L. Gai, Nguyen Thi Kim Thanh

**Affiliations:** 1 International Iberian Nanotechnology Laboratory, 4715-330 Braga, Portugal; 2 Biophysics Group, Department of Physics and Astronomy, University College London, London WC1E 6BT, UK; simone.famiani.15@ucl.ac.uk (S.F.); andreas.sergides.15@ucl.ac.uk (A.S.); 3 UCL Healthcare Biomagnetics and Nanomaterials Laboratories, London W1S 4BS, UK; 4 The York Nanocentre, University of York, York YO10 5DD, UK; leoardo.lari@york.ac.uk (L.L.); david.lloyd@york.ac.uk (D.C.L.); ed.boyes@york.ac.uk (E.D.B.); pratibha.gai@york.ac.uk (P.L.G.); 5 School of Material Science, Japan Advanced Institute of Science and Technology (JAIST), Ishikawa, Kanazawa 923-1292, Japan; mari@jaist.ac.jp (M.T.); shinya@jaist.ac.jp (S.M.)

**Keywords:** Kirkendall effect, in situ oxidation, core-shell

## Abstract

The oxidation of solution-synthesized iron (Fe) and iron carbide (Fe_2_C) nanoparticles was studied in an environmental scanning transmission electron microscope (ESTEM) at elevated temperatures under oxygen gas. The nanoparticles studied had a native oxide shell present, that formed after synthesis, an ~3 nm iron oxide (Fe_x_O_y_) shell for the Fe nanoparticles and ~2 nm for the Fe_2_C nanoparticles, with small void areas seen in several places between the core and shell for the Fe and an ~0.8 nm space between the core and shell for the Fe_2_C. The iron nanoparticles oxidized asymmetrically, with voids on the borders between the Fe core and Fe_x_O_y_ shell increasing in size until the void coalesced, and finally the Fe core disappeared. In comparison, the oxidation of the Fe_2_C progressed symmetrically, with the core shrinking in the center and the outer oxide shell growing until the iron carbide had fully disappeared. Small bridges of iron oxide formed during oxidation, indicating that the Fe transitioned to the oxide shell surface across the channels, while leaving the carbon behind in the hollow core. The carbon in the carbide is hypothesized to suppress the formation of larger crystallites of iron oxide during oxidation, and alter the diffusion rates of the Fe and O during the reaction, which explains the lower sensitivity to oxidation of the Fe_2_C nanoparticles.

## 1. Introduction

Partially pure zero-valent iron nanoparticles have long been used for magnetic and catalytic applications [1]. More recently, they have been explored for biomedical applications, such as contrast enhancement for magnetic resonance imaging (MRI) [2,3], magnetic particle imaging [4] and magnetic hyperthermia [5]. Iron is of particular interest due to its impressive magnetic properties and biocompatibility due to their abundance in the human body [1]. However, iron readily oxidizes to iron oxide under ambient conditions, which degrades its magnetic properties. Therefore, several strategies have been employed to stabilize the iron core for use in applications, such as increasing the size of the iron core and coating the particles to mitigate the oxidation [5,6,7]. One such methodology has been to carbidize the iron, making it less susceptible to oxidation [8].

Iron carbides have been extensively studied as catalysts, such as in the Fischer–Tropsch process to replace noble metal catalysts [9,10,11,12]. Additionally, iron carbide has attracted attention due to its magnetic properties and potential biocompatibility. Iron carbide possesses appealing magnetic properties as it is an iron carbon alloy where iron maintains its metallic nature [13]. The carbon in the crystal structure provides good chemical stability against oxidation, minimizing the drop in magnetization that occurs due to oxidation over time [8]. For this reason, iron carbides have been studied as a theranostic tool for MRI, photothermal therapies and photoacoustic imaging [14,15,16,17]. Chaudret et al. showed that Fe_3_C/Fe_2_C nanoparticles were also promising for magnetic heating during catalysis [18]. 

The carbide is an essential component to minimize the oxidation of the iron nanoparticles and thereby optimize their use in the aforementioned applications. To fully understand how this occurs, a detailed understanding of the oxidation mechanism is required, as developed in this paper. Currently, the studies of iron nanoparticle oxidation have been carried out on the more common amorphous nanoparticles, either ex situ [19], or in situ via wide-angle X-ray scattering [20]. However, a similar exploration has not been carried out on the more biomedically applicable crystalline α-iron nanoparticles or iron carbide nanoparticles.

Ex situ studies of oxidation and reduction reactions have been extensively used to understand the final state of a reaction, but detailed mechanistic information is often hampered by the difficulties posed by trapping or quenching a reaction intermediate without altering it. These studies also have difficulty investigating the reaction of individual particles or grains, tending to study population level differences in the sample. Conversely, in situ experiments can study the exact same sample over time, and follow and observe metastable structures throughout the reaction. One of the most informative techniques for studying oxidation processes in situ transmission electron microscopy (TEM), where individual particles or grains can be followed throughout a reaction. Environmental TEM studies are carried out by injecting gas directly into the column of the microscope localized around the sample, while maintaining the high resolution of the TEM [21]. The sample can then be heated up to the reaction conditions using a heating holder. Such studies have been instrumental to understanding oxidation mechanisms of metal systems [22], as well as for catalytic reaction dynamics [23,24]. In particular, Z-contrast imaging via high-angle annular dark field environmental scanning transmission electron microscopy (HAADF-ESTEM) has been pivotal to studying single atom reactions [25,26] and further increased the ability to readily track oxidation mechanisms and phase changes in situ [27]. 

Herein, we study the oxidation mechanisms of chemically synthesized α-Fe nanoparticles and Fe_2_C nanoparticles with ESTEM.

## 2. Materials and Methods

### 2.1. Materials

1-octadecene (ODE, 90%), hexadecylamine (HDA, 90%), diethyl ether (anhydrous, ≥99.7%), hydrochloric acid (HCl, 37%), oleylamine (OAm, 70%) and octadecylamine (ODA, 70%) were purchased from Sigma Aldrich, Tokyo, Japan. Ethanol (EtOH, 99.5%) was obtained from Nacalai Tesque Inc., Kyoto, Japan. Iron pentacarbonyl (Fe(CO)_5_, >95%), hexane (>96%) and chloroform (CHCl_3_) were obtained from Kanto Chemicals, Tokyo, Japan. All the reagents were used as purchased without any further purification.

### 2.2. Synthesis of Iron Carbide Nanoparticles

A 50 mL 3-neck flask was loaded with 10 mL of ODE, 0.05 mmol of hexadecylammonium chloride (HDA-Cl) and 3 mmol of ODA. HDA-Cl was synthesized following a previously published procedure [28]. The solution was purged with nitrogen for 1 h at 120 °C to remove oxygen, and then the temperature was raised to 180 °C and 0.2 mL of Fe(CO)_5_ was injected into the solution. The solution remained at this temperature for half an hour to form the initial iron nanoparticles and then the temperature was raised to 260 °C for 15 min to form the iron carbide phase. Once the reaction was complete, the heating mantle was removed, and the solution was cooled to room temperature. 

The nanoparticle dispersion was washed via centrifugation with a 1:3 ratio mixture of chloroform:ethanol at 5000 rpm for 3 min. The supernatant was discarded, and the precipitated nanoparticles were redispersed in chloroform. Ethanol was then added for the next centrifugation step, and this was carried out three times in total and the nanoparticles were dispersed in chloroform. A drop of the nanoparticles was then pipetted out and drop-dried onto a Wildfire heating D9 double-tilt chip with a silicon nitride support (DENSsolutions, Delft, The Netherlands) and allowed to dry for the in situ measurements. 

### 2.3. Synthesis of Iron Nanoparticles

Synthesis of Fe NPs was carried out via a previously reported procedure [5]. OAm (0.160 mL, 0.5 mmol), HDA-Cl (0.138 g, 0.5 mmol) and ODE (10 mL) were mixed in a 50 mL 3-neck flask and purged with nitrogen for 1 h at 120 °C. The temperature was increased to 180 °C, and Fe(CO)_5_ was pumped into the solution at a rate of addition of 0.4 mL/h using a KDS100 syringe pump (KD Scientific Inc., Holliston, MA, United States). The total injection time was 25 min for a total injected amount of 0.17 ml of Fe(CO)_5_. The washing procedure and sample preparation for microscopy was the same as for the Fe_2_C nanoparticles.

### 2.4. Environmental Scanning Transmission Electron Microscopy

The ESTEM experiments were carried out on a double aberration-corrected environmental (scanning) TEM (ACE(S)TEM) based on a JEOL 2200FS (JEOL Ltd., Tokyo, Japan) modified by Boyes and Gai [25,26]. The microscope was equipped with gas injection facilities, a differential pumping system and a field emission gun, and operated at 200 kV. The temperature was controlled with a microelectromechanical Wildfire double-tilt heating stage (DENSsolutions, Delft, The Netherlands). The samples were heated for 30 min at 300 °C inside the microscope column in a vacuum before the experiments were carried out to remove organic ligands and prevent carbon build-up during the in situ experiments [29]. No changes were observed during this procedure. After 30 min, the stage temperature was lowered to 20 °C and the high-purity oxygen gas (99.999% from BOC Ltd, Guilford, UK) was introduced into the column, and stabilized at a partial pressure of 2 Pa oxygen within 2 min. When the gas pressure was stable, the temperature was rapidly raised to 200 °C. Time zero was recorded once the reaction system reached 200 °C. The beam was kept blank except during image acquisition (20.4 s exposure) and refocusing (~10 s). The oxidation experiments were run for up to 35 min at 200 °C, and at this point, full oxidation had been achieved for both the nanoparticle systems. The particles were imaged every 1–2 min until 15 min, then 3–5 min until the reaction had been completed. Areas not exposed to the electron beam were imaged after the reaction to compare to the area that was studied and confirm that the behavior was not beam-induced (Supplementary Figure S1). The images were acquired with a pixel size of 0.14 nm, a frame size of 1024 × 1024 pixels and a dwell time of 19.5 μs. Core size, shell size and hollow size measurements were measured from 20 particles at different times in the reaction. 

### 2.5. Scanning Transmission Electron Microscopy

Ex situ high angle annular dark field scanning transmission electron microscopy (HAADF-STEM) and aberration corrected high resolution transmission electron microscopy (HRTEM) was carried out on a double aberration-corrected Titan Themis 60–300 (FEI co., Hillsboro, OR, USA), equipped with an X-FEG gun operating at 200 kV, equipped with an Enfinum GIF and a monochromator. Electron energy loss spectroscopy (EELS) was taken in monochromated STEM with dual EELS and an energy spread of 0.3 eV. The high-resolution EELS spectra were smoothed and then graphed.

Particle size and oxide shell size were measured from a distribution of 100 particles, with the average size and standard deviation calculated.

## 3. Results

### 3.1. Characterization of Fe and Fe_2_C Nanoparticles

**Figure 1:**
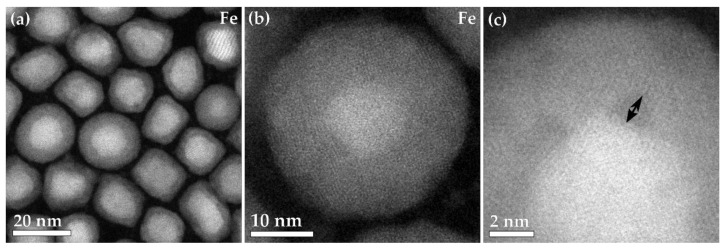
(**a**–**c**) HAADF-STEM images of the Fe. The higher magnification images show the interface between the core and the oxide shell, with black arrows noting the voids formed.

The natively oxidized particles of Fe had an average particle size of 16 nm and a standard deviation of the particle distribution of 2 nm, and the oxide shell had an average shell thickness of 3.1 nm and standard deviation of 0.6 nm (Figure 1a,b). The oxide shell can be readily seen with the duller contrast in the HAADF-STEM image surrounding the brighter Fe core (Figure 1a). The high-resolution image of the Fe nanoparticle (Figure 1c) shows that there are small voids that form at room temperature between the crystalline Fe core and the crystalline Fe_x_O_y_ shell (Figure 1c). The Fe nanoparticles are formed with a crystalline α-Fe bcc core, with a crystalline Fe_x_O_y_ shell formed of either maghemite or magnetite, the full characterization and synthesis of these nanoparticles were reported previously [5]. 

**Figure 2:**
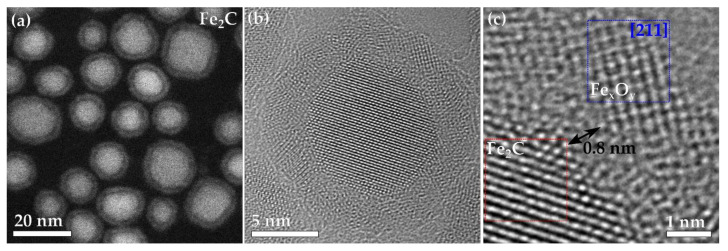
(**a**) HAADF-STEM image of the Fe_2_C nanoparticles. (**b**) Aberration-corrected HRTEM of a whole particle of Fe_2_C. (**c**) A zoomed in area focusing on the Fe_x_O_y_ shell and the void between it and the Fe_2_C core.

The Fe_2_C nanoparticles had an average size of 14 nm and a standard deviation of the particle size distribution of 2 nm, with an average oxide thickness of 1.9 nm and standard deviation of 0.3 nm (Figure 2a). The Fe_2_C nanoparticles had a noticeable void around the entire particle (Figure 2b). The whole shell is made up of small crystallites of ~2 nm in size, with a void space between the core and the shell of ~0.8 nm (Figure 2c), with several areas having a couple of atoms that can be seen joining between the void and the shell. The shell is formed of small crystallites of a Fe_x_O_y_ spinel (maghemite or magnetite), with one crystalline part of the shell shown in Figure 2c, in the blue box, viewed down the [211] zone axis. The Fe_2_C nanoparticles were characterized with X-ray diffraction (Supplementary Figure S2), X-ray photoelectron spectroscopy (Supplementary Figure S3 and Table S1) and Mössbauer spectroscopy (Supplementary Figure S4 and Table S2) to confirm their composition, and the particles’ cores were formed with crystalline Fe_2_C and the shells with small Fe_x_O_y_ crystallites.

### 3.2. ESTEM Imaging of Fe Nanoparticles

**Figure 3:**
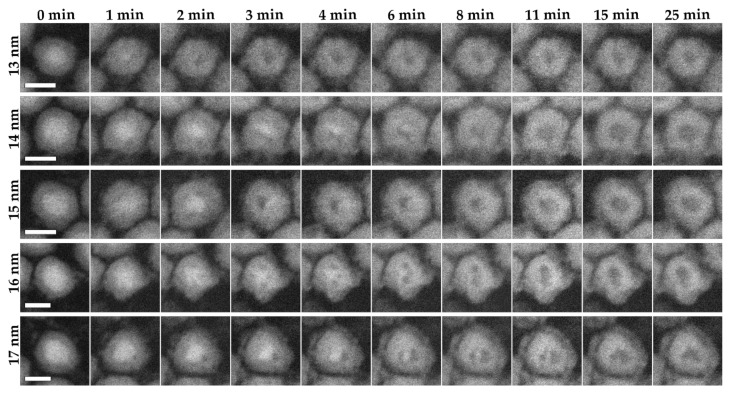
In situ visualization and analysis of dynamic oxidation of Fe nanoparticles in HAADF-ESTEM at 200 °C with 2 Pa oxygen. The figure shows nanoparticles with increasing particle sizes (13–17 nm) over a period of 25 min. The images have a 10 nm scale bar.

The ESTEM experiments were carried out first with the Fe nanoparticles at 200 °C in 2 Pa oxygen. Several particles of different sizes (13, 14, 15, 16, 17 nm) were followed (Figure 3). In all cases, the small core started oxidizing from one or several void areas that formed between the iron core and the oxide shell (Figure 3, 1 and 2 min). The oxidation rapidly progressed from the voids and continued until the remaining core area was only attached to one part of the shell. Finally, the entire core disappeared, leaving a hollow space in the center of the particle. The time taken for the particles to fully oxidize is based on their total size, which corresponds to their core size. The smallest (13 nm) particle oxidized the fastest, within 3 min, while the largest (17 nm) particle took 25 min to fully oxidize. The oxidation of the iron occurred from the native oxide shell of ~3.5 nm, which rapidly increased in thickness to the final shell thickness of ~5.5 nm once the central core had disappeared. The hollow that remained once the core was fully oxidized was smaller than the initial core size, with the initial core sizes being 6.6, 8.3, 8.0, 10.2 and 10.0 nm, and the hollows being 4.7, 6.2, 7.0, and 6.1 nm after 25 min for the 13, 14, 15, 16 and 17 nm particles respectively. This indicated that the shell also fills some of the hollow space as it oxidizes.

### 3.3. ESTEM Imaging of Fe_2_C Nanoparticles

**Figure 4:**
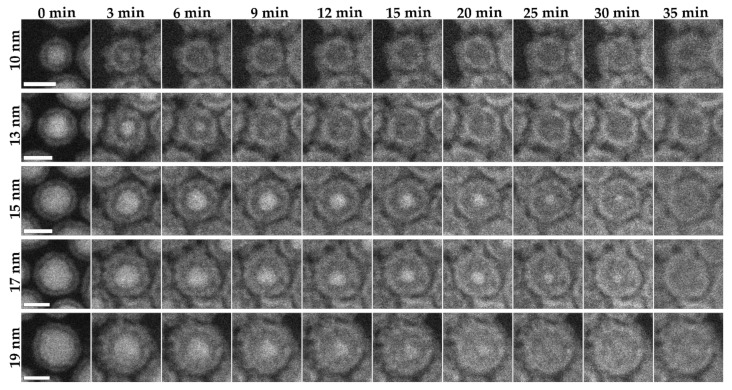
In situ visualization and analysis of dynamic oxidation of Fe_2_C nanoparticles in HAADF-ESTEM at 200 °C with 2 Pa oxygen. The figure shows nanoparticles with increasing particle sizes (10–19 nm) over a period of 35 min. The images have a 10 nm scale bar.

Several different particle sizes of Fe_2_C nanoparticles (10, 13, 15, 17, 19 nm) were studied via ESTEM experiments at 200 °C in 2 Pa oxygen (Figure 4). In all cases, the oxidation occurred symmetrically, with the initial oxide core decreasing in size from the center of the particle until it entirely disappeared. The oxide shell grew rapidly during the initial stages of the oxidation reaction, from ~2 nm at 0 min before the reaction started to ~3 nm at 3 min at 200 °C in 2 Pa oxygen, and finishing at ~3.6 nm once the core was completely oxidized. The particles take longer to oxidize roughly based on the size of the Fe_2_C, with the 15 and 17 nm particles taking the longest at 35 min and the 10 nm particle fully oxidizing within 5 min. The 19 nm particle oxidized slightly faster than the 15 and 17 nm particles, showing some variability in the oxidation rate outside of the pure particle core. The oxide shell that formed shortly after the reaction started was not uniform in size with larger and smaller sections of the oxide shell. The size of the hollow space that was left upon full oxidation of the particle was observed to be similar in size to the initial core that was oxidized, with a core size of 6.4, 8.6, 11.4, 11.8 and 14 nm at 0 min and hollow size of 6.2, 8.2, 10.8, 11.6 and 13.3 nm after 35 min for the 10, 13, 15, 17, and 19 nm particles respectively. This indicates that the majority of the iron carbide adds to the surface of the shell during the reaction, with a final total particle size of 11.5, 15.5, 16.5, 17.8 and 21.6 nm for the 10, 13, 15, 17, and 19 nm particles respectively. The electron diffraction pattern taken after the reaction had completed confirmed that Fe_x_O_y_ is the final product of the reaction (Supplementary Figure S5).

**Figure 5:**
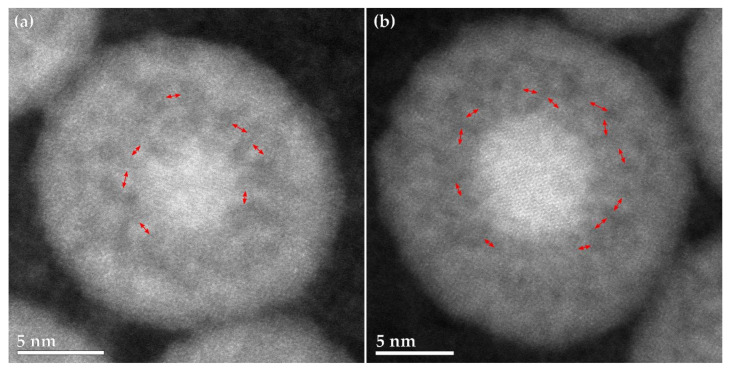
(**a**,**b**) High-resolution HAADF-STEM image of partially oxidized nanoparticles of Fe_2_C. Red double-sided arrows indicate the bridge material crossing the void.

High-resolution images of two partially oxidized Fe_2_C particles showed bridges of the material surrounding the core in all areas. The bridges (as shown by the red arrows in Figure 5) were all observed to be leading from the core to the outer shell and were approximately 1 nm in size. There were void spaces that formed between the different bridges, leaving the void area between the core and the shell made up of a mixture of small voids and small bridges evenly distributed around the core. It should also be noted that some of the iron material will be left within the void area due to the bridges and would remain once the particle is fully oxidized.

**Figure 6:**
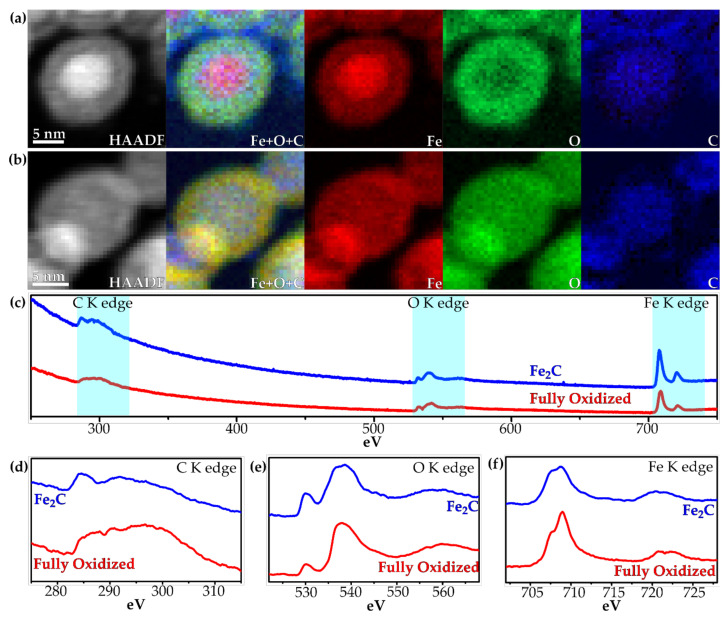
EELS spectrum image maps of (**a**) the Fe_2_C and (**b**) the fully oxidized Fe_2_C, showing the HAADF, overlayed map, Fe, O and C. All maps were generated from the windows in the EELS spectra shown in (**c**). (**d**–**f**) High-resolution EELS spectra of the C K edge, O K edge and Fe K edge, respectively, of the Fe_2_C and fully oxidized nanoparticles.

Monochromated EELS was carried out with a ~0.3 eV energy spread. Spectrum imaging EELS maps were carried out with a 0.25 eV dispersion to simultaneously collect the signals for the carbon, oxygen and iron K edges. The maps from the spectrum images from a Fe_2_C nanoparticle before the reaction are shown in Figure 6a, and for a hollow fully oxidized nanoparticle in Figure 6b, and both are extracted from the spectra shown in Figure 6c. The Fe_2_C nanoparticle shows the Fe signal from both the core and the shell, while the O signal is only seen in the shell and the C signal in the core, as expected for Fe_2_C nanoparticles. After oxidation, the Fe signal and O signal are both seen in the hollow shell, however the C signal is still seen within the core filling the area where the shell is not. Higher resolution spectra with an energy dispersion of 0.05 eV were taken of the C, O and Fe K edges for an area of Fe_2_C nanoparticles and fully oxidized nanoparticles. The C K edge for the Fe_2_C nanoparticles shows a distinctive peak at 285 eV and a lower broader peak spanning from 288 to 304 eV, with its highest position at 292 eV. These results are indicative of the π* and σ* features of carbon, and have been previously shown for iron carbide [30]. After oxidation, the C K edge is largely without distinctive features, with the main features at 286, 290 and 296.5 eV (Figure 6d). For the O K edge, the Fe_2_C nanoparticles have three major features: a smaller peak at 530.3 eV, a larger peak at 538.4 eV and a broader peak at 558.6 eV, while in the oxidized particles the peaks are seen at 530.3 eV, a larger peak at 537.9 eV and a broad peak at 561.5 eV (Figure 6e). The form of the peaks has been reported for iron oxide, with the secondary and broader peaks shifting to higher energy for Fe^3+^ [31]. Finally, for the Fe K edge, there are two major peaks of Fe shown, the L_2_ and L_3_ peak, and both of these have two different components. For the Fe_2_C nanoparticles, this is at 707.7 and 720.3 eV, with a 1.1 and 1.3 eV splitting, respectively (Figure 6f), and is similar to previous reports for iron carbides [30], while for the oxidized nanoparticles, the peaks are seen at 707.5 and 720.9 eV with a splitting of 1.4 and 1.5 eV, respectively (Figure 6f). The shorter initial peak to the secondary peak and the larger splitting have been previously reported for Fe_2_O_3_ [30,31].

**Figure 7:**
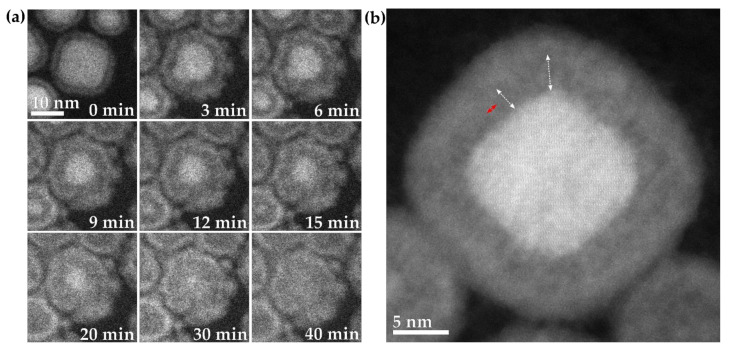
(**a**) In situ visualization and analysis of dynamic oxidation of a nanocube of Fe_2_C in HAADF-ESTEM at 200 °C with 2 Pa oxygen. (**b**) High-resolution HAADF-STEM image of a partially oxidized nanocube of Fe_2_C@Fe_x_O_y_, with the white double-sided arrows indicating the void section and the red double-sided arrow indicating the bridge material.

Although the iron carbide nanoparticles were typically spherical in shape, there were two other shapes formed in the sample, cubes and elongated rods. First off, studying the Fe_2_C nanocubes, it was observed that at the start of the oxidation, the cubic core started adopting a spherical shape as the core rounded during oxidation (Figure 7a, 3–9 min). Once the initial rounding had occurred, the oxidation preceded identically to the spherical Fe_2_C nanoparticles (Figure 7a, 12–40 min). High-resolution imaging of a partially oxidized cube showed an initial degree of rounding in the corners and also showed the same oxide bridges that were observed for the spherical nanoparticles (Figure 7b).

**Figure 8:**
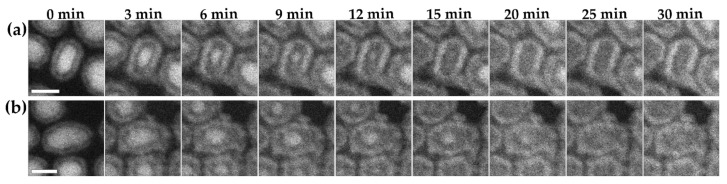
(**a**,**b**) In situ visualization and analysis of dynamic oxidation of two different rods of Fe_2_C in HAADF-ESTEM at 200 °C with 2 Pa oxygen. The figure shows two elongated nanoparticles (**a**,**b**) over a period of 30 min. The images have a 10 nm scale bar.

Elongated rods of Fe_2_C were also studied. It was observed that the oxidation occurred predominantly along the long axis of the nanoparticles. In Figure 8a, an elongated nanoparticle is shown that is 16 nm long and 10 nm wide. The particle oxidized more rapidly from the two rounded ends than the relatively flatter sides, becoming nearly spherical in shape near the center of the oxidizing rod at 9 min. In the case of the rod shown in Figure 8b, the rod is 20 nm long and 13 nm wide at the widest part, and the oxidation again occurred rapidly from both ends, until the central unoxidized core was in the wider part of the nanoparticulate rod (at around 9 min) and was roughly spherical. After this, a more symmetrical oxidation occurred until the Fe_2_C core was fully oxidized. Such asymmetric oxidation has been seen previously with shape-controlled nanoparticles, which showed that the oxidation occurred rapidly until the central unoxidized core area was approximately spherical in nature, and that the further from a spherical configuration a particle was, the more rapid its initial oxidation [32].

## 4. Discussion

**Figure 9:**
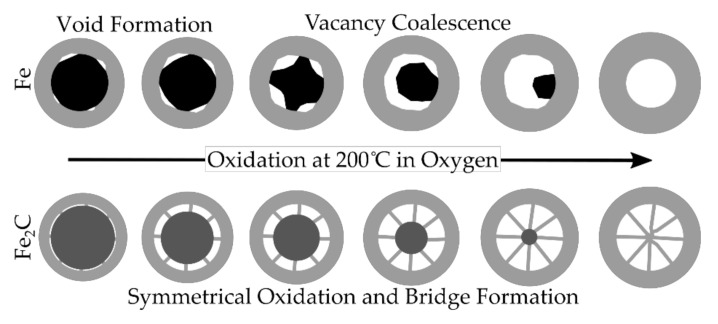
Schematic of the observed oxidation pathways of Fe and Fe_2_C indicating the initial void formation and coalescence that occurs during the oxidation of Fe, and the oxide bridge formation and growth of the shell that occurs with the Fe_2_C.

The iron nanoparticles oxidized from the formation of initial voids and then asymmetrically with the exposed void areas increasing in size and coalescing until the iron core was fully oxidized. In contrast, the iron carbide nanoparticles oxidized symmetrically with the core shrinking in the center of the nanoparticles and the shell increasing in size outside of the shell until the particles were fully oxidized (Figure 9). One interesting difference between the two mechanisms is that although the shell thickness increased in size for both materials, for the Fe, the shell thickness increased more and the hollow space left behind was smaller, whereas for the Fe_2_C, the hollow space was almost identical to the initial size of the core and the increase in the shell thickness was smaller. Some of the material stayed as part of the oxide bridges that formed, whereas the majority of the material added to the size of the shell outside of where the initial core was. 

The Fe nanoparticles represent a typical oxidation mechanism that has been observed previously with other metallic nanoparticles, such as Ni [22,33] and NiCr alloys [34], studied via in situ TEM. In these cases, the metallic systems have been observed to begin oxidizing often from a single void and progressing from that initial void. In the case of NiFe, one, two or more initial voids were seen, and coalescence occurred as the oxidation progressed [35]. In these cases, defects are observed forming between the larger oxide grains that form in the shell, and these are hypothesized to create channels that allow the metal cations to diffuse through the shell to the source of oxygen. For pure α-iron nanoparticles oxidized ex situ, these multiple voids forming around the core have previously been reported [36], and as shown in this study, will coalesce during oxidation into a single hollow space. These mechanisms occur via what is known as the nanoscale Kirkendall effect, and the void formation at the surface between the metal core and shell occurs due to differential diffusion rates, where the metal diffuses outwards faster than the oxygen diffuses inwards.

The iron carbide nanoparticles also behave in a same manner, but the void formation occurs symmetrically, where bridges of the final material form during the reaction. This has been observed for the nanoscale Kirkendall effect with the formation of oxides, sulfides and selenides [37]. The difference here points to a larger imbalance of the diffusion kinetics between the oxygen and the iron, which is what gives rise to the hollowing. In the case of iron carbide, the oxygen reacts with the Fe and the C, and should lead to the formation of Fe_3_O_4_/Fe_2_O_3_ and CO_2_/CO, respectively [38]. However, in this case, the EELS analysis indicated that a large amount of carbon remained trapped within the core, and the presence of this carbon could cause the diffusion imbalance and essentially inhibit any diffusion of oxygen into the structure, forcing the oxide shell to be formed and grown only outside of the shell area. The carbon has also been previously reported to adhere to defects in iron oxide and alter its nucleation [38]. In this study, the Fe_2_C was also observed to have smaller Fe_x_O_y_ grains in its shell, and the Fe_x_O_y_ bridges could be inhibited by the carbon or formed as an iron oxycarbide.

Previous studies with amorphous iron ex situ have shown a similar behavior to the iron carbide nanoparticles, and in one article the authors also note some presence of carbon in the iron particles [19]. An in situ study with wide-angle X-ray scattering of amorphous iron nanoparticles in solution indicated that even though the nanoparticles looked to have a similar void between the core and the shell, this was actually made up of small initial voids that grew in three dimensions to surround the iron core by void coalescence until complete oxidation had occurred [20]. In the case of the Fe_2_C nanoparticles, the void surrounding the particle would also be a network of coalesced voids with bridges connecting the core and the shell. The iron cations could be oxidized and transported along the material bridges to the oxide shell where the oxidation occurs.

In this study, the Fe and Fe_2_C had underlying crystallographic differences, with Fe having a cubic crystal structure and Fe_2_C having a hexagonal one. The change in crystal structure upon oxidation would cause more rearrangement to occur and the formation of the interfacial voids, along with the inhibiting effect of the carbon. Conversely, Fe and its oxides are all cubic, and during oxidation, a stable (010)Fe//(220)Fe_3_O_4_ or (010)Fe//(002)Fe_3_O_4_ interface can form [39], and strain and diffusion will lead to void formation in a few initial places based on the spherical nature of the particle. Once the void formation occurs, the oxidation occurs via mass transport in the areas where the shell is in contact with the core, leading to the growth of the void areas until finally, void coalescence occurs. With the carbide, the formation of numerous small voids leads to bridges where the mass transport of the Fe ions is transported, and these bridges remain within the structure even after full oxidation occurs.

## 5. Conclusions

The formation of the iron carbide nanoparticles greatly altered the oxidation mechanism of the nanoparticles. Whereas the iron nanoparticles oxidized through a more classic mechanism occurring through void formation and then coalescence, the iron carbide nanoparticles oxidized through the formation of ~1 nm stable oxide bridges and a symmetrical shrinking of the carbide core over the course of the reaction, while the Fe ions diffused out of the core to the growing Fe_x_O_y_ shell, leaving the carbon behind in the hollow core.

## Data Availability

The data presented in this study are available in the article and supplementary information.

## Supplementary Materials

The following are available online at https://www.mdpi.com/1996-1944/15/4/1557/s1, Figure S1: HAADF-STEM images of areas not exposed to the electron beam during the reactions. Figure S2: XRD pattern of the Fe_2_C nanoparticles. Figure S3: Fe 2p core level XPS for the Fe_2_C nanoparticles. Figure S4: Room temperature ^57^Mössbauer spectrum of the Fe_2_C nanoparticles. Figure S5: Electron diffraction of the Fe_2_C before the reaction, and after showing the particles were oxidized to iron oxide (Fe_x_O_y_). Table S1: Fit parameters for the Fe 2p core level XPS of the Fe_2_C nanoparticles. Table S2: Best fit parameters of the ^57^Mossbauer spectrum of Fe_2_C nanoparticles.
