# Supplementary material for: Environmental STEM Study of the Oxidation Mechanism for Iron and Iron Carbide Nanoparticles
Source: Materials (Basel). 2022 Feb 18;15(4):1557. doi: 10.3390/ma15041557 (PMC8877599; doi:10.3390/ma15041557)
Supplement: Supplementary file 1 [file materials-15-01557-s001.zip › materials-1541693-supplementary.pdf]

materials

MDPI

Supplementary Materials

Environmental STEM Study of the Oxidation Mechanism for Iron and Iron Carbide Nanoparticles

Alec P. LaGrow [1,*], Simone Famiani [2,3], Andreas Sergides [2,3], Leonardo Lari [4], David C. Lloyd [4], Mari Takahashi [5], Shinya Maenosono [5], Edward D. Boyes [4], Pratibha L. Gai [4] and Nguyen Thi Kim Thanh [2,3,*]

[1] International Iberian Nanotechnology Laboratory, 4715-330 Braga, Portugal
[2] Biophysics Group, Department of Physics and Astronomy, University College London, London WC1E 6BT, UK; simone.famiani.15@ucl.ac.uk (S.F.); andreas.sergides.15@ucl.ac.uk (A.S.)
[3] UCL Healthcare Biomagnetics and Nanomaterials Laboratories, London W1S 4BS, UK
[4] The York Nanocentre, University of York, York YO10 5DD, UK; leoardo.lari@york.ac.uk (L.L.); david.lloyd@york.ac.uk (D.C.L.); ed.boyes@york.ac.uk (E.D.B.); pratibha.gai@york.ac.uk (P.L.G.)
[5] Japan Advanced Institute of Science and Technology (JAIST), School of Material Science, Ishikawa 923-1292, Japan; mari@jaist.ac.jp (M.T.); shinya@jaist.ac.jp (S.M.)
* Correspondence: alec.lagrow@inl.int (A.P.L.); ntk.thanh@ucl.ac.uk (N.T.K.T.)

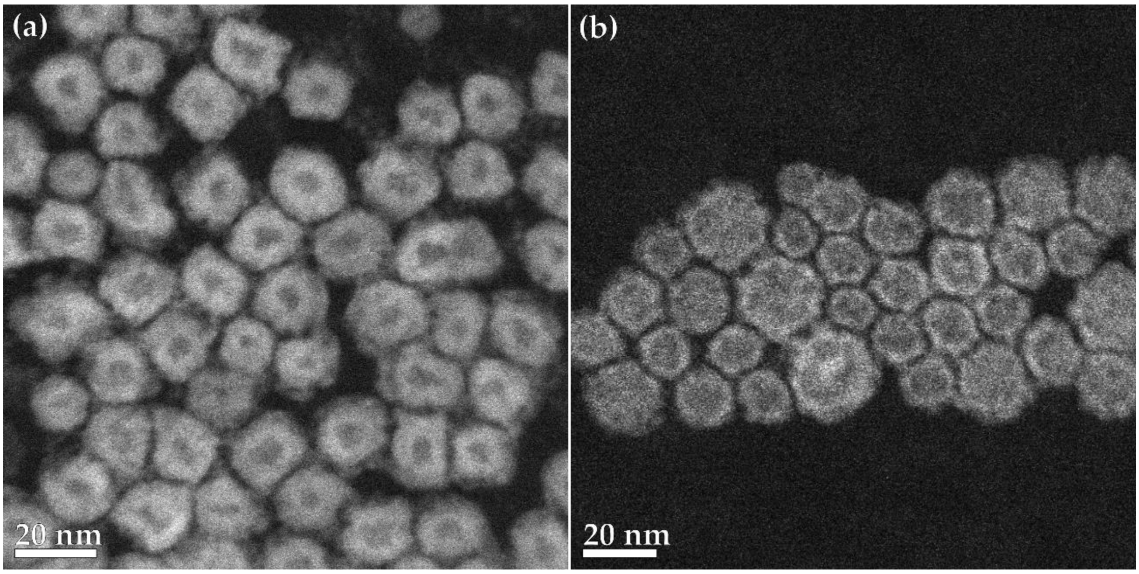

Figure S1. HAADF-STEM images of areas not exposed to the electron beam during reaction of (a) the Fe nanoparticles after 25 minutes of reaction time and (b) the Fe2C nanoparticles after 35 minutes of reaction time at 200 C in 2 Pa O_2.

Characterization of the Iron Carbide Nanoparticles

The nanoparticle samples were studied with a X-ray diffractometer (XRD, Rigaku MiniFlex600) using a Cu Kα source (λ = 1.5418 Å). The XRD pattern shows that the nanoparticles phase is Fe_2C without noticeable peaks from Fe or Fe_3O_4 (**Figure S1**).

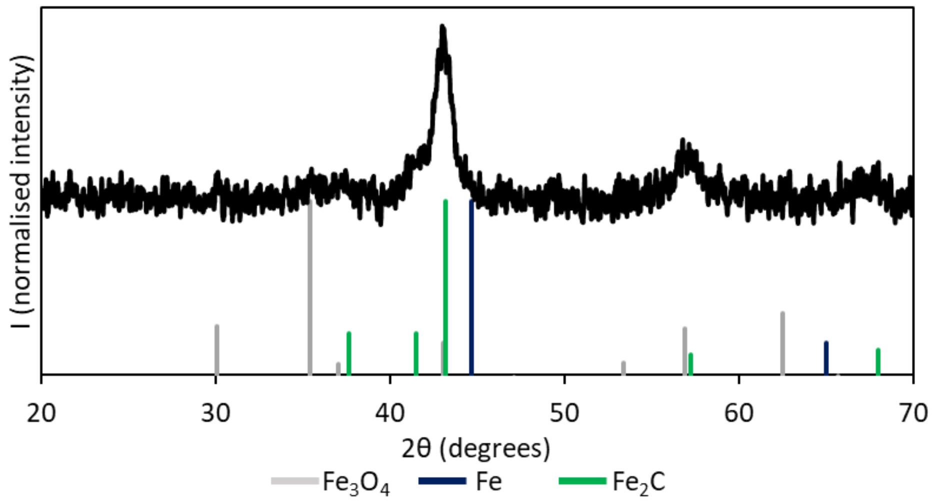

Figure S2. XRD pattern of the Fe_2C nanoparticles. Coloured guide bars are shown for Fe_3O_4 (PDF No. 01-079-0418), Fe (PDF No. 00-006-0696), Fe_2C (PDF No. 00-036-1249).

X-ray photoelectron spectroscopy (XPS) was performed on a high-performance XPS system (Shimadzu Kratos, Axis-Ultra DLD) using Al Kα radiation on the Fe_2C. The Fe 2p spectra for both materials are shown in **Figure S2**. The Fe 2p spectra for the Fe_2C nanoparticles were fit using six different peaks; Fe^0, Fe^{2+}, Fe^{3+}, high-binding energy surface structures, satellite peaks, and an extra species appearing in the spectrum at around 704 eV (Table S1).[19]

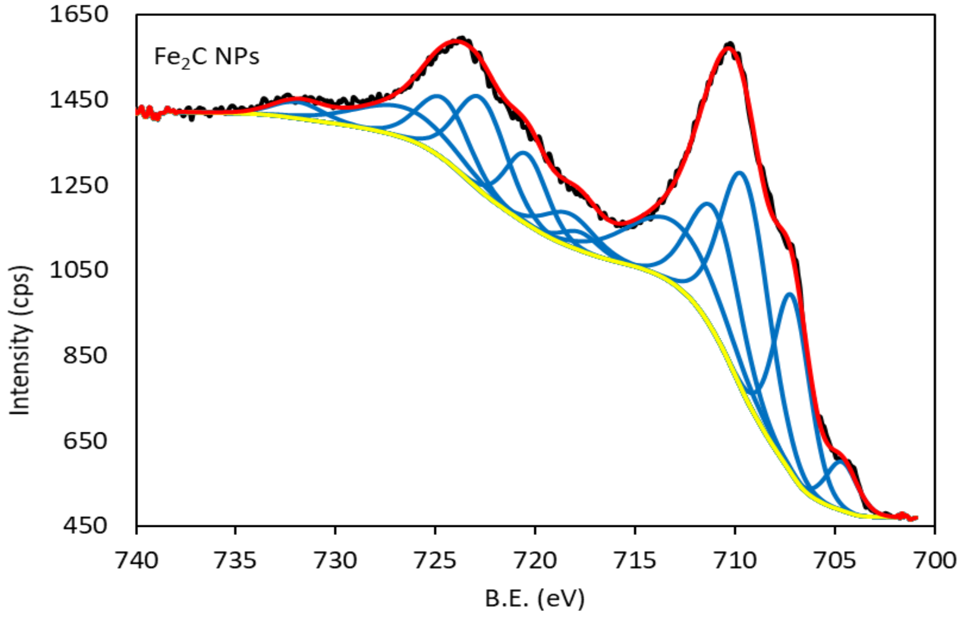

Figure S3. Fe 2p core level XPS for the Fe_2C nanoparticles. Black, yellow, blue, and red curves represent raw data, Shirley background, deconvoluted peaks and the sum of the components, respectively.

Table S1. Fit parameters for the Fe2p core level XPS of the Fe_2C nanoparticles.

Component	BE $2p_{3/2}$ [eV] (FWHM)	BE $2p_{1/2}$ [eV] (FWHM)	BE ($2p_{1/2}$-$2p_{3/2}$) [eV]	Spin-orbit intensity ratio	Proportion (%)
Fe-C	704.6 (1.8)	717.7 (2.2)	13.1	2.2	6.1
Fe^0	707.1 (2.1)	720.4 (2.4)	13.3	2.2	25.7
Fe^{2+}	709.5 (2.8)	722.7 (2.9)	13.2	2.2	42.0
Fe^{3+}	711.0 (3.0)	724.6 (3.0)	13.6	2.0	26.2
Fe surface	713.0 (5.5)	726.5 (5.7)	13.5	2.0	-
Fe satellite	718.3 (3.0)	731.9 (3.0)	13.6	2.0	-

In the case of Fe_2C nanoparticles, the Fe^0 species is 26 %. The peak at 704.6 eV has a lower binding energy than Fe^0 suggesting that the species is closer to the metallic state rather than an oxidized one, which is attributed to Fe-C.[20]

To confirm the different iron phases present in the final structure, Mössbauer spectroscopy was performed on Fe_2C NPs. **Figure S3** shows the ^{57}Fe Mössbauer Spectrum on the Fe_2C nanoparticles and the relative fit. The parameters used for the fitting are listed in **Table S2**. 58 mg of the Fe_2C nanoparticle powder sample was placed in a mortar with ~100 mg pure sucrose powder and mixed to form a solid dispersion. The powder was pressed between two plastic disks to form a coin-shaped absorber of 2.1 cm diameter. Measurements were performed at room temperature using a SeeCo W302 constant acceleration drive with a triangular reference signal. The source was driven at a velocity scale of 12 mm/s. Data were accumulated in 1024 channels. The spectrum was fitted with Recoil software and was folded relative to a 10 μm thick α-Fe foil.

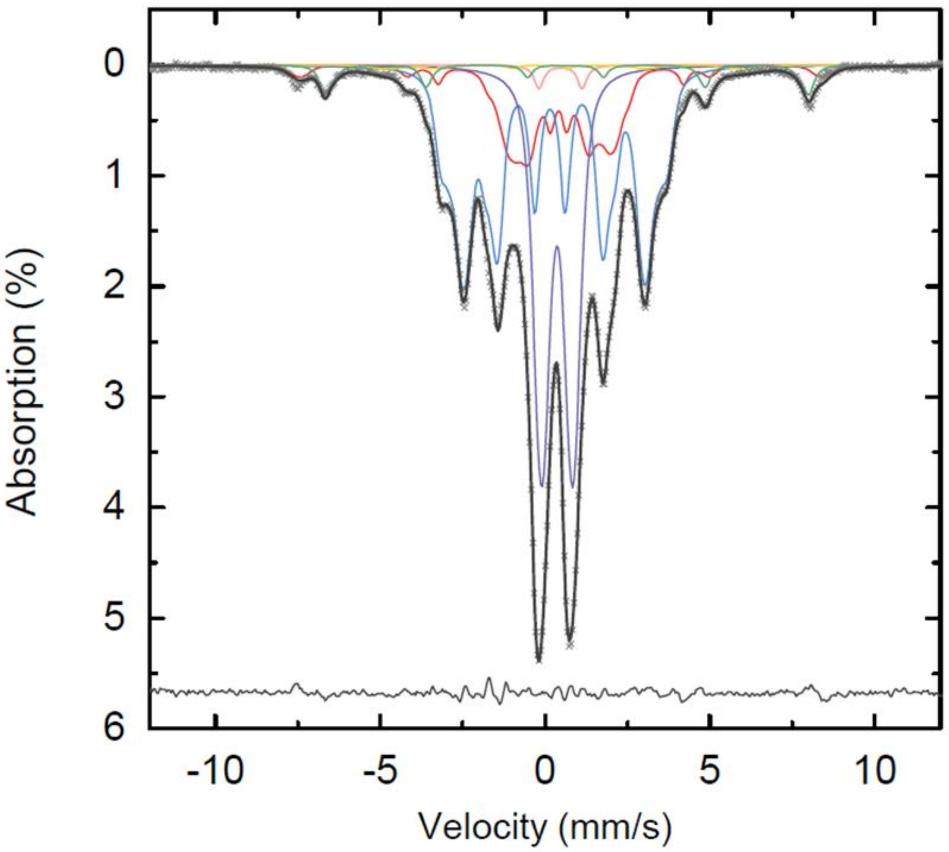

Figure S4. Room temperature ^{57}Mössbauer spectrum of the Fe_2C nanoparticles. The fits are shown using a superposition of two Voigtian doublets and four Voigtian sextets (the coloured lines). The residual to the measured data is indicated by the grey line at the bottom.

The spectrum is a superposition of at least 4 sextets and a quadrupole doublet. Voigtian line shapes were used to represent a Gaussian distribution of Lorentzian lines,

which arise from a range of sizes, shapes, chemical compositions and crystallinity in the sample.

Table S2. Best fit parameters of the [57]Mossbauer spectrum of Fe_2C nanoparticles.

δ (mm/s)	Q (mm/s)	ε (mm/s)	H (KOe)	w3 (mm/s)	Relative area (%)		Assigned species
0.366 ± 0.002	0.9443 ± 0.0007	-	-	0.17	30.3		Amorphous Fe^{3+}
0.47 ± 0.03	1.31 ± 0.05	-	-		1.2		
0.217 ± 0.002	-	0.065 ± 0.002	170; σ = 10		70.6	44.5	Fe_2C
			197; σ = 2		7.2		
			213; σ = 6		22.2		
0.447 ± 0.006	-	0.03 ± 0.03	96; σ = 16		80.0	19.1	Mixture of ferric oxide and Fe-C alloy
			230; σ = 0.1		9.2		
			490; σ = 13		10.8		
0.64 ± 0.01	-	0.02 ± 0.02	442; σ = 9		30.0	3.8	Ferric oxide
			455; σ = 3		70.0		
0.2 ± 0.1	-	0.4 ± 0.1	335; σ = 30		1.1		α-Fe

Electron diffraction of the Fe_2C nanoparticles were carried out before the reaction and afterwards in the microscope (Figure S4). After the reaction the pattern matches that of Fe_xO_y (maghemite or magnetite).

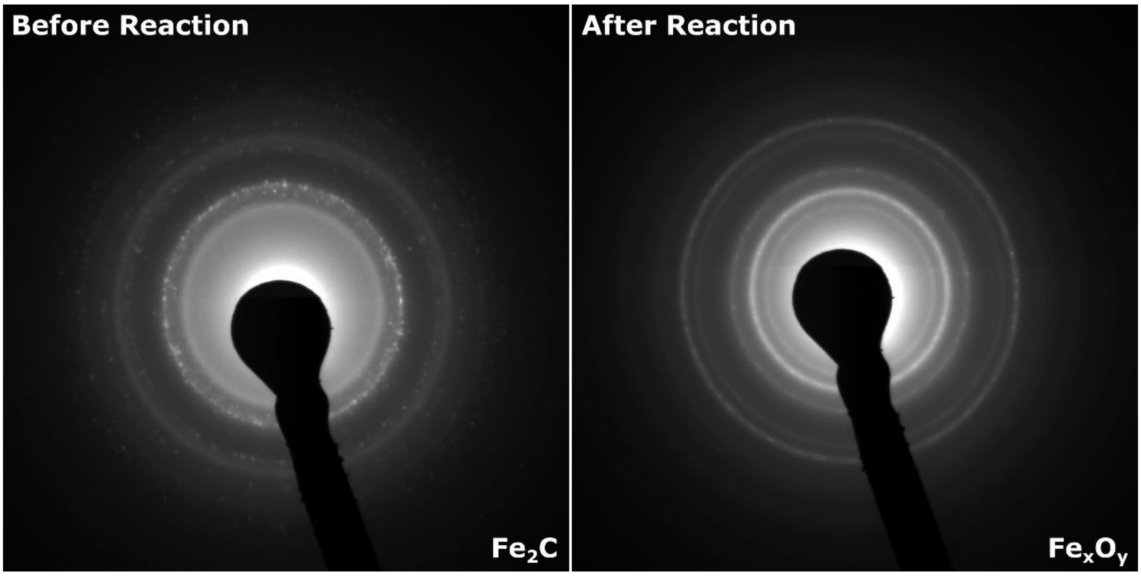

Figure S5. Electron diffraction of the Fe_2C before the reaction, and after showing the particles were oxidized to iron oxide (Fe_xO_y).
